# Zileuton Improves Memory Deficits, Amyloid and Tau Pathology in a Mouse Model of Alzheimer’s Disease with Plaques and Tangles

**DOI:** 10.1371/journal.pone.0070991

**Published:** 2013-08-07

**Authors:** Jin Chu, Jin-Guo Li, Domenico Praticò

**Affiliations:** Department of Pharmacology, Temple University School of Medicine, Philadelphia, Pennsylvania, United States of America; Cleveland Clnic Foundation, United States of America

## Abstract

The 5-lipoxygenase (5LO) enzyme is widely distributed within the central nervous system. Previous works showed that this protein is up-regulated in Alzheimer’s disease (AD), and plays an active role in the development of brain amyloidosis in the APP transgenic mice. In the present paper, we studied the effect of its pharmacological inhibition on the entire AD-like phenotype of a mouse model with plaques and tangles, the 3×Tg mice. Compared with mice receiving placebo, the group treated with zileuton, a specific 5LO inhibitor, manifested a significant improvement of their memory impairments. The same animals had a significant reduction in Aβ levels and deposition, which was secondary to a down-regulation of the γ-secretase pathway. Additionally, while total tau levels were unchanged for both groups, zileuton-treated mice had a significant reduction in its phosphorylation state and insoluble forms, secondary to a decreased activation of the cdk5 kinase. These data establish a functional role for 5LO in the pathogenesis of the full spectrum of the AD-like phenotype and represent the successful completion of the initial step for the preclinical development of 5LO inhibitors as viable therapeutic agents for AD.

## Introduction

The enzyme 5-lipoxygenase (5LO) catalyzes the conversion of arachidonic acid to 5-hydroxy-peroxy-eicosatetraenoic acid (5-HPETE) and subsequently to 5-hydroxy-eicosatetraenoic acid (5-HETE), which can be then metabolized into different leukotrienes [1]. The 5LO is widely expressed in the central nervous system (CNS), where it localizes mainly in neuronal cells. Its presence has been documented in various regions of the brain, including hippocampus, where significant changes in its levels have been associated with aging [2]. Since aging is one of the strongest risk factors for developing sporadic Alzheimer’s disease (AD), this pathway has been involved in its pathogenesis. Thus, 5LO protein immunoreactivity is increased in hippocampi of AD patients versus controls, and polymorphism of the 5LO gene promoter influences the age of onset of the disease [3], [4]. Further, 5LO gene knockout or its pharmacologic inhibition resulted in a significant reduction in brain amyloid β (Aβ) pathology of the Tg2576 mice, a model of AD-like amyloidosis [5], [6].

More recently wed showed that 5LO gene transfer or targeted gene disruption result in worsening or amelioration of the AD-like phenotype in a transgenic mouse model with plaques and tangles, the 3×Tg mice [7], [8]. However, in order for these results to have a translational value, it still remains to be established whether 5LO pharmacological inhibition would results in an improvement of their memory impairments and AD neuropathologies.

With this goal in mind, in the current study we chronically administered the 3×Tg mice with a selective and orally available 5LO inhibitor, i.e., zileuton [9]. At the end of the study we observed that compared with mice receiving vehicle, the group treated with zileuton had a significant improvement of their memory impairments. The same mice had a significant reduction in the amount of Aβ formed and deposited in their brains, which was secondary to significant reduction in the γ-secretase pathway. Additionally, we observed that treated mice had a significant decrease in the phosphorylation of tau, which was associated with a reduction in the cdk-5 kinase activation.

## Methods

### Mice and Treatments

All animal procedures were approved by Temple University Institutional Animal Care and Usage Committee (protocol #4137), and in accordance with the Guide for the Care and Use of Laboratory Animals of the National Institute of Health. The 3×Tg mice harboring a mutant APP (KM670/671NL), a human mutant PS1 (M146V) knockin and tau (P301L) transgenes were used in this study. They were kept in a pathogen-free environment, on a 12-hour light/dark cycle and had access to food and water ad libitum. A total of eighteen mice were available for this study, with 5 female and 4 male mice per group. Starting at 2–3 months of age, mice were randomized to receive zileuton (200 mg/L) (n = 9) or vehicle (n = 9) in their drinking water for 10 months until they were 12–13 month-old. At this age time-point, they underwent behavioral testing and two weeks later sacrificed. Considering that each mouse drinks in average 3–4 ml/day of water, the final concentration of the active drug was approximately 0.6–0.8 mg/day. During the study, mice in both groups gained weight regularly, and no significant differences in weight were detected between the two groups. No macroscopic effect on the overall general health was observed in the animals receiving the active treatment. Post-mortem examination showed no sign of macroscopic pathology in any of the organs considered (i.e., spleen liver, thymus, ileum). After sacrifice, animals were perfused with ice-cold 0.9% Phosphate Buffered saline (PBS), brain removed and dissected in two hemihalves by mid-sagittal dissection. One half was immediately stored at −80°C for biochemistry assays, the other immediately immersed in 4% paraformaldehyde in 0.1 M PBS (pH 7.6) overnight for immunohistochemistry studies.

### Behavioral Tests

All animals were pre-handled for 3 days prior testing, they were tested in a randomized order, and all tests conducted by an experimenter blinded to the treatments.

### Fear-conditioning

Two weeks before sacrifice, fear conditioning experiments were performed following methods previously described [7], [8]. Briefly, tests were conducted in a conditioning chamber (19×25×19 cm) equipped with black methacrylate walls, transparent front door, a speaker and grid floor (Start Fear System; Harvard Apparatus). On day one, mice were placed into the conditioning chamber and allowed free exploration for 2 min in the white noise (65 Db) before the delivery of the conditioned stimulus (CS) tone (30 s, 90 Db, 2000 Hz) paired with a foot-shock unconditioned stimulus (US; 2 s, 0.6 mA) through a grid floor at the end of the tone. A total of 3 pairs of CS-US pairing with a 30 s inter trial interval (ITI) were presented to each animal in the training stage. The mouse was removed from the chamber 1 min after the last foot-shock and placed back in its home cage. The contextual fear-conditioning stage started 24 h after the training phase when the animal was put back inside the conditioning chamber for 5 min with white noise only (65 dB). The animal’s freezing responses to the environmental context were recorded. The tone fear-conditioning stage started 2 h after the contextual stage. The animal was placed back to the same chamber with different contextual cues, including white wall, smooth metal floor, lemon extract drops, and dimmed yellow light condition. After 3 min of free exploration, the mouse was exposed to the exactly same 3 CS tones with 30 s ITI in the training stage without the foot-shock and its freezing responses to the tones were recorded. One minute after the last tone, the mouse was brought back to the home cage.

### Y-maze

The Y-maze apparatus consisted of three arms 32 cm (long) ×10 cm (wide) with 26-cm walls (San Diego Instruments, San Diego, CA). Testing was always performed in the same room and at the same time to ensure environmental consistency as previously described [7], [8]. Briefly, each mouse was placed in the center of the Y-maze and allowed to explore freely through the maze during a 5-min session. The sequence and total number of arms entered were video recorded. An entry into an arm was considered valid if all four paws entered the arm. An alternation was defined as three consecutive entries in three different arms (i.e. 1, 2, 3 or 2, 3, 1, etc). The percentage alternation score was calculated using the following formula: Total alternation number/total number of entries-2)*100. Furthermore, total number of arm entries was used as a measure of general activity in the animals. The maze was wiped clean with 70% ethanol between each animal to minimize odor cues.

### Morris Water Maze

The apparatus used was a white circular plastic tank (122 cm in diameter) with walls 76 cm high, filled with water maintained at 22° +/−2°C, which was made opaque by the addition of a nontoxic white paint, and inside had a removable, square (10 cm in side length) Plexiglas platform. The tank was located in a test room containing various prominent visual cues. Before the first trial of the first session, the mouse was placed for 10 seconds on the platform. Mice were trained to swim to the platform submerged 1.5 cm beneath the surface of the water and invisible to the mice while swimming. The platform was located in a fixed position, equidistant from the center and the wall of the tank. Mice were subjected to four training trials per day (inter-trial interval, 15 minutes). During each trial, mice were placed into the tank at one of four designated start points in a random order. Mice were allowed to find and escape onto the submerged platform. If they fail to find the platform within 60 seconds, they were manually guided to the platform and allowed to remain there for 10 seconds. Mice were trained to reach the training criterion of 20 seconds (escape latency). To control for overtraining, probe trials were run for each group, both as soon as they reached group criterion and after all groups had reached criterion.

Mice were assessed in the probe trial 24 hours after the last training session and consisted in a 60-second free swim in the pool without the platform. Each animal’s performance was monitored using the Any-Maze™ Video Tracking System (Stoelting Co.) which provided data for the acquisition parameters (latency to find the platform and distance swam) and the probe-trial parameter (number of entries to the target platform zone and time in quadrants).

### Biochemical Analyses

Mouse brain homogenates were sequentially extracted first in RIPA for the Aβ soluble fractions and then in formic acid (FA) for the Aβ insoluble fractions as previously described [5]–[8]. Aβ1-40 and Aβ1-42 levels were assayed by a sensitive sandwich ELISA kits (WAKO Chem., Richmond, VA). Analyses were always performed in duplicate and in a coded fashion.

### Sarkosyl Insolubility Assay

The assay for insoluble tau was performed as previously described [10]. Briefly, ultracentrifugation and sarkosyl extraction (30 min in 1% sarkosyl) was used to obtain soluble and insoluble fractions of tau from brain homogenates. Insoluble fractions were washed one time with 1% sarkosyl, then immunoblotted with the HT-7 antibody.

### Western Blot Analyses

RIPA extracts from brain homogenates were used for western blot analyses as previously described [5]–[8]. Samples were electrophoresed on 10% Bis–Tris gels or 3–8% Tris–acetate gel (Bio-Rad, Richmond, CA), according to the molecular weight of the target molecule, transferred onto nitrocellulose membranes (Bio-Rad, Richmond, CA), and then incubated with appropriate primary antibodies as indicated in Table 1. After three washings with T-TBS, membranes were incubated with IRDye 800CW or IRDye 680CW-labeled secondary antibodies (LI-COR Bioscience, Lincoln, NE) at 22°C for 1 h. Signals were developed with Odyssey Infrared Imaging Systems (LI-COR Bioscience, Lincoln, NE). Beta-actin was always used as internal loading control.

**Table 1:** Antibodies used in this study.

Antibody	Immunogen	Host	Application	Source
5-LO	Human 5-Lipoxygenase aa 442–590	Mouse	WB	BD Transduction
4G8	aa 18–22 of human beta amyloid (VFFAE)	Mouse	IHC	Covance
APP	aa 66–81 of APP {N-terminus}	Mouse	WB	Millipore
BACE-1	aa human BACE (CLRQQHDDFADDISLLK)	Rabbit	WB	IBL
ADAM10	aa 732–748 of human ADAM 10	Rabbit	WB	Millipore
PS-1	aa around valine 293 of human presenilin 1	Rabbit	WB	Cell Signaling
Nicastrin	aa carboxy-terminus of human Nicastrin	Rabbit	WB	Cell Signaling
APH-1	Synthetic peptide from hAPH-1a	Rabbit	WB	Millipore
Pen-2	aa N-terminal of human and mouse Pen-2	Rabbit	WB	Invitrogen
sAPPα	Synthetic peptide of the C-terminal part of Human sAPPα (DAEFRHDSGYEVHHQK)	Rabbit	WB	Cell Signaling
sAPPβ	Synthetic peptide of the C-terminal part of human sAPPβ-sw (ISEVNL)	Rabbit	WB	Cell Signaling
CTFs	a synthetic peptide [(C)KMQQNGYENPTYKFFEQMQN] corresponding toamino acids 751–770 of human precursor protein (APP),conjugated to KLH	Rabbit	WB	Santa Cruz
HT-7	aa 159–163 of human tau	Mouse	WB, IHC	Pierce
AT-8	Peptide containing phospho-S202/T205	Mouse	WB	Pierce
AT-180	Peptide containing phospho-T231/S235	Mouse	WB	Pierce
AT-270	Peptide containing phospho-T181	Mouse	WB	Pierce
PHF-13	Peptide containing phospho-Ser396	Mouse	WB, IHC	Cell Signaling
PHF-1	Peptide containing phospho-Ser396/S404	Mouse	WB, IHC, IF	Dr. P. Davies
GFAP	aa spinal cord homogenate of bovine origin	Mouse	WB, IHC	Santa Cruz
CD45	Mouse thymus or spleen	Rat	IHC	BD Pharmingen
GSK3α/β	aa 1–420 full length GSK-3β of Xenopus origin	Mouse	WB, IF	Millipore
p-GSK3α/β	aa around Ser21 of human GSK-3a.	Rabbit	WB	Cell Signaling
JNK2	aa of human JNK2	Rabbit	WB	Cell Signaling
SAPK/JNK	aa of recombinant human JNK2 fusion protein	Rabbit	WB	Cell Signaling
Phospho-SAPK/JNK	aa Thr183/Tyr185 of human SAPK/JNK	Mouse	WB	Cell Signaling
Cdk5	aa C-terminus of Cdk5 of human origin	Rabbit	WB, IF	Santa Cruz
P35/25	aa C-terminus of p35/25 of human origin	Rabbit	WB, IF	Santa Cruz
Actin	aa C-terminus of Actin of human origin	Goat	WB	Santa Cruz

### Immunohistochemistry

Immunostaining was performed as reported previously by our group [7], [8], [10], [11]. Briefly, serial coronal sections were mounted on 3-aminopropyl triethoxysilane (APES)-coated slides. Every eighth section from the habenular to the posterior commissure (8–10 sections per animal) was examined using unbiased stereological principles. The sections for testing Aβ were deparaffinized, hydrated, pretreated with formic acid (88%) and subsequently with 3% H_2_O_2_ in methanol. The sections used for testing HT7, PHF-1, PHF-13, and GFAP, CD45 were deparaffinized, hydrated and subsequently with 3% H_2_O_2_ in methanol, and then antigen retrieved with citrate (10 mM) or IHC-Tek Epitope Retrieval Solution (IHC world). Sections were blocked in 2% fetal bovine serum before incubation with primary antibody overnight at 4°C (Wako Chemicals, Richmond, VA). After washing, sections were incubated with biotinylated anti-mouse IgG (Vector Lab, Burlingame, CA) and then developed by using the avidin-biotin complex method (Vector Lab, Burlingame, CA) with 3,3′-diaminobenzidine (DAB) as a chromogen. Light microscopic images were used to calculate the area occupied by Aβ-immunoreactivity, the cell densities of GFAP and CD45 using the software Image-ProPlus for Windows version 5.0 (Media Cybernetics). The threshold optical density that discriminated staining from background was determined and kept constant for all quantifications.

### Data Analysis

Data analyses were performed using SigmaStat for Windows version 3.00. Statistical comparisons were performed by Unpaired Student’s t-test or the Mann-Whitney rank sum test when a normal distribution could not be assumed. Values in all figures and table represent mean ± S.E.M. Significance was set at p<0.05.

## Results

### Pharmacologic Blockade of 5LO Ameliorates Cognition in 3×Tg Mice

**Figure 1:**
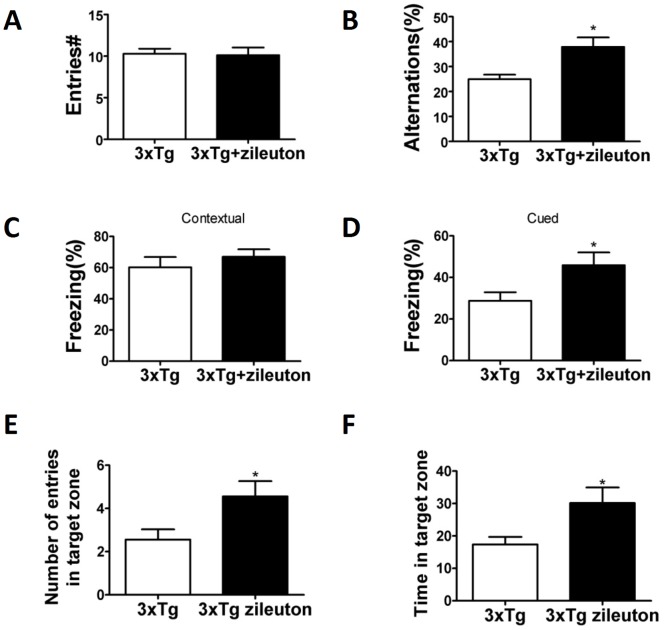
Chronic administration of zileuton ameliorates behavioral deficits of 3×Tg-AD mice. **A**. Number of total arm entries for 3×Tg mice receiving zileuton or vehicle. **B**. Percentage of alternations between 3×Tg mice receiving zileuton or vehicle treatment (*p<0.05). **C**. Contexual fear memory response in 3×Tg mice receiving zileuton or controls. **D**. Cued fear memory response in 3×Tg mice receiving zileuton or control (*p<0.05). Values represent mean ± SEM; *p<0.05; n = 9 3×Tg; n = 9 3×Tg zileuton. **E**. Number of entries to the target platform zone for 3×Tg mice treated with zileuton or vehicle. **F**. Time in the target platform zone for 3×Tg mice treated with zileuton or vehicle (*p<0.05).

In the Y-maze, 3×Tg mice receiving zileuton and the group treated with placebo did not show any significant difference in the total number of arm entries, suggesting that there were no differences in their general motor activity (Figure 1A). However, the number of alternation for the mice receiving the drug was increased when compared with their controls, as shown by their higher percentage values (Figure 1B). In the fear conditioning test, both groups did not manifest any difference during the training phase (data not shown). However, 3×Tg mice receiving zileuton had a significantly higher freezing time in the cued recall paradigm than the ones receiving placebo (Figure 1D), but no differences were observed in the contextual recall (Figure 1C). Mice were also tested in the reference spatial memory function by using the Morris water maze. In these studies, we performed visible platform training followed by hidden platform testing with four probe trials per day. All mice in each group were able to reach the training criterion within 4 days and were similarly proficient swimmers (data not shown). However, in the probe trial 3×Tg mice treated with zileuton had significant increase in the number of entries to the target zone and the time spent in the target zone when compared to controls (Figure 1E, 1F). By contrast, there were no changes for the latency to first entry to the target zone, the distance travelled until first entry in the target zone, and time spent in the opposite target zone (data not shown).

### Pharmacological Blockade of 5LO Reduces Aβ Levels and Deposition via the γ-Secretase Pathway

Two weeks after the behavioral testing were completed, animals were sacrificed and their brains harvested for biochemistry and immunohistochemistry analyses.

**Figure 2:**
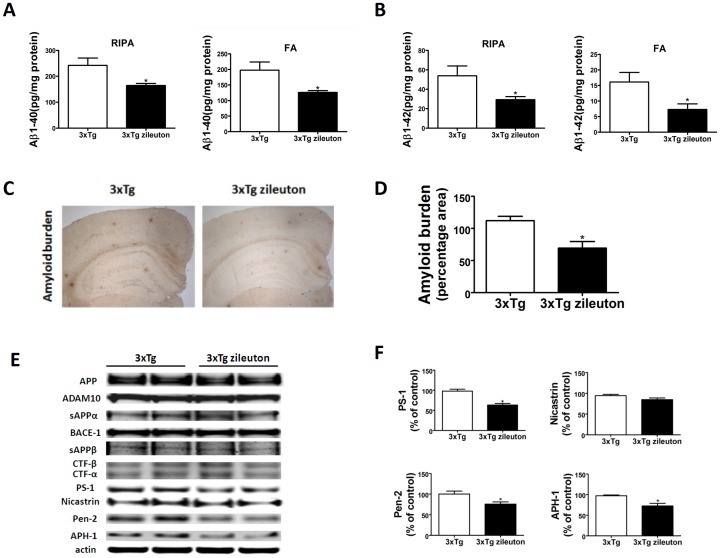
Pharmacologic blockade of 5LO decreases Aβ peptides levels and deposition in the 3×Tg mice via the γ-secretase pathway. A, B. RIPA-soluble (RIPA) and formic acid extractable (FA) Aβ1-40 and Aβ1-42 levels in cortex of 3×Tg receiving zileuton or vehicle were measured by sandwich ELISA. (n = 9 for control, and n = 9 for zileuton; *p<0.05). C. Representative sections of brains from 3×Tg mice receiving zileuton or placebo (control) immunostained with 4G8 antibody. D. Quantification of the area occupied by Aβ immunoreactivity in brain of 3×Tg mice receiving zileuton or placebo (n = 3 control, and n = 3 zileuton; *p<0.05). E. Representative western blots of APP, ADAM-10, BACE-1, sAPPs, CTFs, PS1, Nicastrin, Pen-2, and APH-1 in the cortex of 3×Tg mice receiving zileuton or vehicle. F. Densitometric analyses of the immunoreactivities to the antibodies shown in the previous panel. (n = 4, *p<0.05). Values represent mean ± SEM.

As expected for their age, 12–13 month-old 3×Tg mice on placebo showed elevated levels of both soluble (RIPA extractable) and insoluble (Formic acid extractable) Aβ1-40 and Aβ1-42 in their brain, which were significantly reduced in mice receiving zileuton (Figure 2A). To determine the effect on brain Aβ deposition, the areas occupied by 4G8-immunopositive reactions were analyzed by immunohistochemistry. Comparison of the Aβ immuno-positive areas between placebo and zileuton-treated group revealed a statistically significant reduction of the amyloid burden in the treated mice (Figure 2B, 2C).

To understand the mechanism responsible for this effect on Aβ, we investigated the metabolism of its precursor, the Aβ precursor protein (APP). To this end, steady state levels of APP, α-secretase (ADAM-10), β-secretase (BACE-1), and the four components of the γ-secretase complex were assayed by western blot analysis. As shown in figure 2, levels of APP, ADAM-10, and BACE-1 were unchanged, by contrast, we observed that mice receiving zileuton had a significant decrease in the steady state levels of three of the four components of the γ-secretase complex, PS1, Pen-2 and APH-1.

### Pharmacological Blockade of 5LO Modulates Tau Metabolism via cdk-5 Kinase

**Figure 3:**
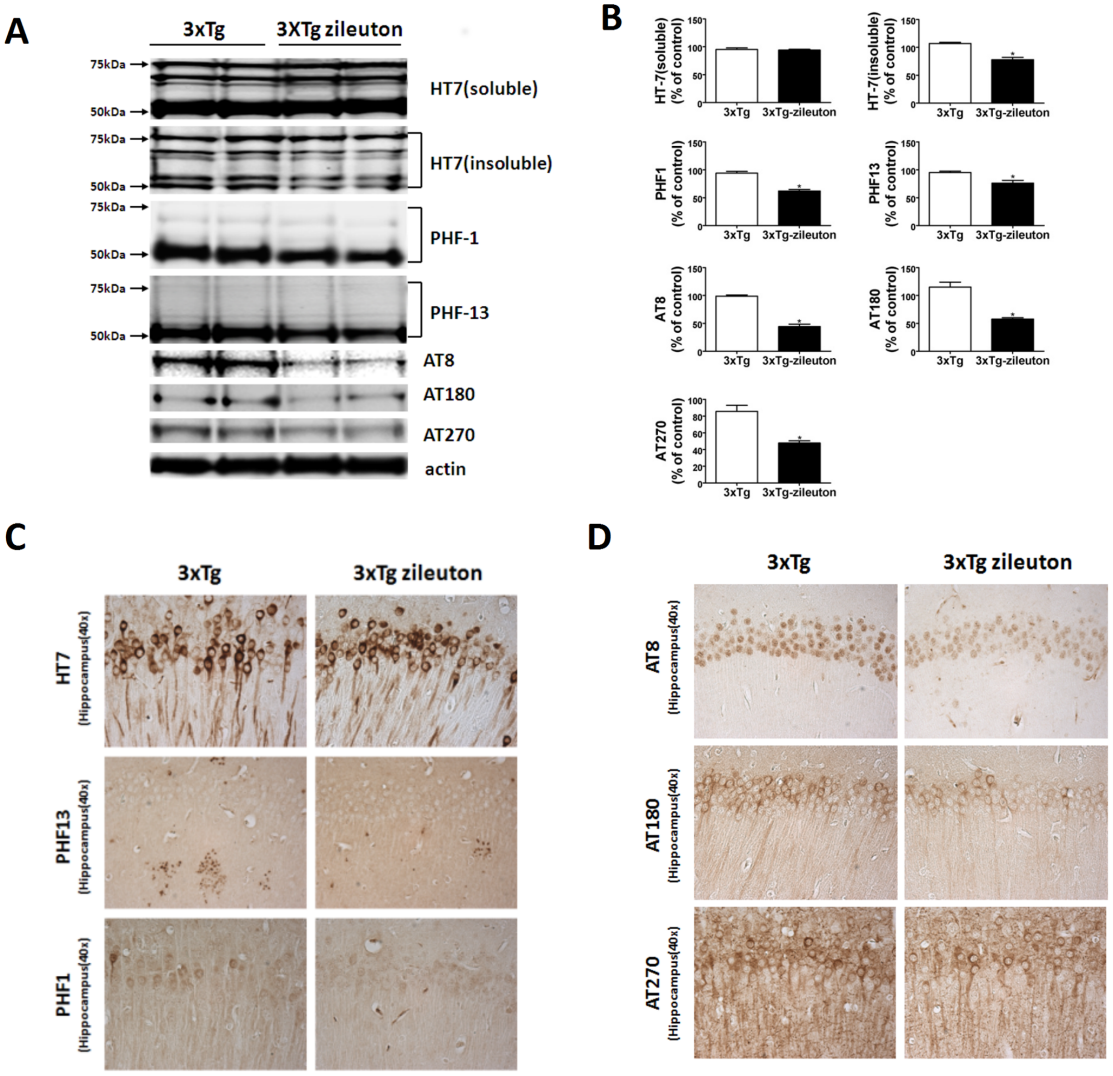
Pharmacologic blockade of 5LO reduces tau phosphorylation in the 3×Tg mice. **A**. Representative western blots of soluble and insoluble total tau (HT7), phosphorylated tau at residues S396 (PHF13), S202/T205 (AT8), T231/S235 (AT180), at T181 (AT270), and S396/S404 (PHF-1) in brain homogenates from 3×Tg mice receiving zileuton or vehicle assayed by western blot analyses. **B**. Densitometric analyses of the immunoreactivities to the antibodies shown in the previous panel (*p = 0.05). **C**, **D**. Representative sections of brains from 3×Tg mice receiving zileuton or vehicle (control) immunostained with HT7, PHF1, PHF13, AT8, AT180, AT270 antibodies.

Next, we examined the effect of zileuton on tau levels and metabolism in the two groups of mice. Compared with controls, mice chronically treated with the drug had a significant decrease in the insoluble tau fraction while there was no change in total soluble tau levels between the two groups (Figure 3 A, B). In addition, we found that the same animals manifested a significant reduction in its phosphorylated forms at different epitopes: S396, as recognized by the antibody PHF-13; S396/404, as recognized by the antibody PHF-1; S202/T205, as recognized by the antibody AT8; T231/S235, as recognized by the antibody AT180; and T181, as recognized by the antibody AT270 (Figure 3A, B).

**Figure 4:**
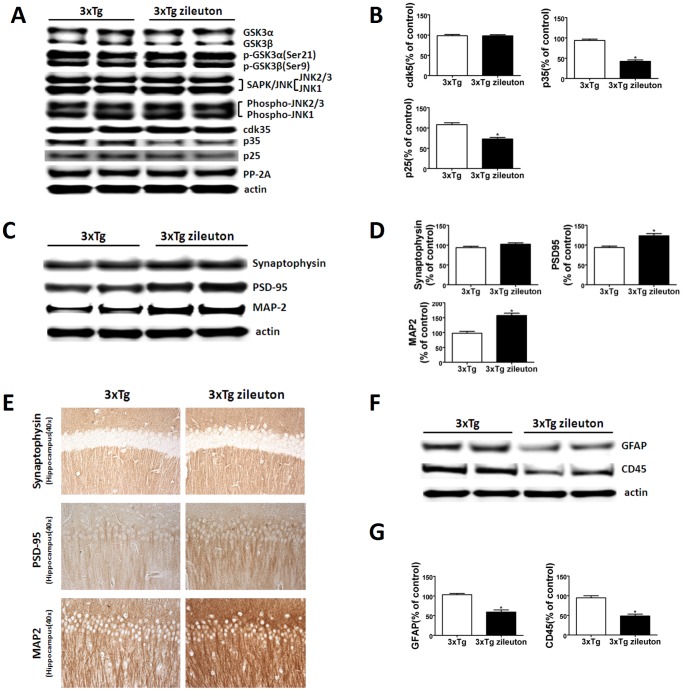
Pharmacologic blockade of 5LO modulates tau metabolism, ameliorates synaptic integrity and decreases neuroinflammation in the 3×Tg mice. A. Representative western blots of GSK3α, GSK3β, p-GSK-3α, p-GSK-3β, JNK2, SAPK/JNK, p-JNK2/3, p-JNK1, cdk5, p35, and p25 in brain homogenates of 3×Tg mice treated with zileuton or vehicle. B. Densitometric analyses of the immunoreactivities to the antibodies shown in the previous panel (*p<0.05). C. Representative western blot analyses of synaptophysin (SYP), post-synaptic density protein 95 (PSD-95), and microtubule-associated protein 2 (MAP2) in brain homogenates of 3×Tg mice receiving zileuton or control. D. Densitometric analyses of the immunoreactivities to the antibodies shown in the previous panel (n = 3 per group) (*<0.05). E. Representative sections of brains from 3×Tg mice receiving zileuton or vehicle (control) immunostained with synaptophysin, PDS-95, and MAP2 antibodies. F. Representative western blots of GFAP and CD45 in brain homogenates of 3×Tg mice treated with zileuton or vehicle. G. Densitometric analyses of the immunoreactivities to the antibodies shown in the previous panel (*p<0.05). Values represent mean ± SEM.

Consistent with the immunoblot results, immunohistochemical staining demonstrated decreased dendritic accumulations of the same phosphorylated isoforms (Figure 3C, D) in the brains of mice treated with zileuton, while there was no significance difference between the two groups for total tau immunoreactivity (Figure 3C). To investigate the mechanism involved in this effect on tau phosphorylation, next we examined some of the kinases which are considered major regulators of its post-translational modifications. No differences in the levels of total or phosphorylated GSK-3α and GSK-3β, JNK2, and total and phosphorylated SAPK/JNK were observed between the two groups of mice (Figure 4A, B). By contrast, we found that brains of 3×Tg mice treated with zileuton had a significant reduction of the cdk5 kinase pathway, as demonstrated by a significant decrease in both the p25 and p35 fragments (Figure 4A, B).

### Pharmacological Blockade of 5LO Modulates Synaptic Integrity and Neuroinflammation

Since tau pathology has been correlated with the severity of dementia and memory impairments for which synaptic integrity is an important factor, next we investigated whether pharmacological blockade of 5LO had any effect on this aspect of the AD-like phenotype. As shown in figure 4C–E, we observed that compared with controls, steady state levels of three distinct synaptic proteins synaptophysin, post-synaptic protein-95 (PDS-95) and MAP-2 were significantly increased in mice receiving zileuton. In addition, we observed that compared with controls these mice had a significant decrease in GFAP and CD45 immunoreactivities suggesting an inhibition of astrocytes and microglia cell activation, respectively (Figure 4F, G).

## Discussion

The data presented in this study demonstrate that pharmacologic blockade of 5-LO significantly ameliorates behavioral deficits, reduces brain Aβ deposition, tau phosphorylation and improves synaptic integrity in the 3×Tg mouse model of AD.

Taken together these data establish a functional role for 5LO in the pathogenesis of the full spectrum of the AD-like phenotype and represent the successful completion of the initial step for the preclinical development of 5LO inhibitors as viable therapeutic agents for AD.

Previous studies have supported a biologic link between 5LO and AD, by showing that this enzymatic pathway acts as an endogenous modulator of Aβ in vivo and in vitro [5], [6], [12]. More recently, we have demonstrated also that its genetic manipulation modulates the AD-like phenotype of a mouse model with plaques and angles, the 3×Tg [7], [8]. However, whether these findings have potential to be translated into a pre-clinical scenario, where drugs blocking this protein could be used as novel therapeutic tools for AD remains to be fully established. With this goal in mind, we designed the pharmacological studies presented in the current paper. Thus, using zileuton, an orally available selective and specific 5LO inhibitor, we first demonstrated that this therapeutic approach results in a significant amelioration of their memory impairments. Next, we showed that the same animals manifested a significant reduction in their brain amyloidosis. In an effort to elucidate the mechanisms responsible for the Aβ reduction in the mice receiving zileuton, we assessed the steady-state levels of APP and the levels of three most important proteases involved in its processing, i.e., α-, β- and γ-secretase, which ultimately results in formation Aβ peptides. We found that total APP, BACE-1 and ADAM-10 protein levels were unaltered by the drug, suggesting that the in vivo biological effect is not mediated by an effect on the Aβ precursor or on its α- or β-cleavage proteolytic pathways. By contrast, we observed that three of the four components of the γ-secretase complex were significantly reduced by this treatment confirming the hypothesis that this proteolytic pathway is specifically influenced by the active treatment [12].

Beside Aβ pathology, AD is also characterized by the presence of abundant intracellular neurofibrillary tangles, which are formed mainly by the hyperphosphorylated microtubule-associated protein tau [13]. In the current study, we showed that while pharmacological blockade of 5LO did not alter level of total soluble tau, it significantly reduced the phosphorylation levels of several epitopes, which have been implicated in the development of neurofibrillary tangles [14]. To elucidate the mechanism for the zileuton-dependent reduction of tau phosphorylation, we assayed several putative kinases which are considered major regulators of its post-translational modifications. Among them we measured total and activated forms of GSK-3, JNK2, and SAP/JNK kinases, but no significant differences were detected between the two groups. By contrast, we found that 5LO pharmacological blockade affected specifically the cdk-5 kinase pathway whose activation is regulated by its binding to activator protein p35 and p25, a cleaved product of p35 [15], [16]. Thus, while mice receiving zileuton had total levels of cdk-5 unchanged, compared with controls they showed a significant reduction in both p35 and p25 levels.

Further supporting the role that hyperphosphorylated tau plays in modulating the development of tau pathology, we also observed a significant reduction in its insoluble forms, which have been directly implicated in memory dysfunction and synaptic pathology [17], [18]. Consistent with these and the improved behavioral data, we observed that 3×Tg receiving zileuton had a significant increase in several markers of synaptic integrity (PSD-95 and MAP-2) suggesting an amelioration of synaptic function in the treated animals.

Our study demonstrates the pleiotropic role that 5LO plays in the development of AD pathogenesis by influencing all major aspects of the disease phenotype. It represents the successful completion of the initial step for the preclinical development of 5LO inhibitors as viable therapeutic agents for AD with disease-modifying capacity. This fact is extremely important since so far treatment opportunities for AD have been rather challenging, and as result research in the field has moved more and more toward prevention and away from treatment, leaving five million of AD patients in this country with little hope for new effective drugs. For this reason our results have significant translational value and may offer new therapeutic hope and opportunity in a relatively short time-frame considering that the drug we used in our study is already FDA approved for asthma [19].

## References

[1] Radmark O, Werz O, Steinhilber D, Samuelsson B (2007) 5-Lipoxygenase: regulation of expression and enzyme activity. Trends Biochem Sci 32: 332–341. 17576065 10.1016/j.tibs.2007.06.002

[2] Chinnici CM, Yao Y, Praticò D (2007) The 5-lipoxygenase enzymatic pathway in the mouse brain: young versus old. Neurobiol. Aging 28: 1457–1462. 10.1016/j.neurobiolaging.2006.06.007 16930777

[3] Ikonomovic MD, Abrahamson EE, Uz T, Manev H, Dekosky ST (2008) Increased 5-Lipooxygenase immunoreactivity in hippocampus of patients with Alzheimer’ diseases. J Histochem Cytochem 56: 1065–1073. 18678882 10.1369/jhc.2008.951855 PMC2583907

[4] Qu T, Manev R, Manev H (2001) 5-Lipoxygenase (5-LOX) promoter polymorphism in patients with early-onset and late-onset Alzheimer’s disease. J. Neuropsychiatry Clin Neurosci 13: 304–305. 11449041 10.1176/jnp.13.2.304

[5] Firuzi O, Zhuo J, Chinnici CM, Wisniewski T, Praticò D (2008) 5-Lipoxygenase gene disruption reduces amyloid-β pathology in a mouse model of Alzheimer’s disease. FASEB J 22: 1169–1178. 17998412 10.1096/fj.07-9131.com PMC2698428

[6] Chu J, Praticò D (2011) Pharmacological blockade of 5-Lipoxygenase improves the amyloidotic phenotype of an Alzheimer’s disease transgenic mouse model. Am J Pathol 178 (4): 1762–1769. 10.1016/j.ajpath.2010.12.032 PMC3078454 21435457

[7] Chu J, Giannopoulos PF, Ceballos-Diaz C, Golde TE, Praticò D (2012) 5-Lipoxygenase gene transfer worsens memory, amyloid and tau brain pathologies in a mouse model of Alzheimer disease. Ann Neurol 72: 442–454. 23034916 10.1002/ana.23642 PMC3464917

[8] Giannopoulos PF, Chu J, Joshi YB, Sperow M, Li JG, et al.. (2013) Gene knockout of 5-lipoxygenase rescues synaptic dysfunction and improves memory in the triple-transgenic model of Alzheimer’s disease. Mol Psych. 2013 Mar 12 [Epub ahead of print]. 10.1038/mp.2013.23 PMC3688674 23478745

[9] Riccioni G, DiIlio C, Conti P, Theoharides TC, D’Orazio N (2004) Advances in therapy with anti-leukotriene drugs. Ann Clin Lab Sci 34: 379–387. 15648777

[10] Yoshi BY, Chu J, Praticò D (2012) Stress hormone leads to memory deficits and altered tau phosphorylation in a mouse model of Alzheimer’s disease. J Alz Dis 31(1): 167–176. 10.3233/JAD-2012-120328 PMC3882896 22531419

[11] Yang H, Zhuo J, Chu J, Chinnici C, Praticò D (2010) Amelioration of the Alzheimer’s disease phenotype by absence of 12/15-lipoxygenase. Biol Psych 68 (10): 922–929. 10.1016/j.biopsych.2010.04.010 20570249

[12] Chu J, Praticò D (2011) 5-Lipoxygenase as an endogenous modulator of amyloid beta formation in vivo. Ann Neurol\ 69: 34–46, 2011. 10.1002/ana.22234 PMC3051361 21280074

[13] Gong CX, Iqbal K (2008) Hyperphosphorylation of microtubule-associated protein tau: A promising therapeutic target for Alzheimer disease. Curr Med Chem 15: 2321–2328. 18855662 10.2174/092986708785909111 PMC2656563

[14] Martin L, Latypova X, Terro F (2011) Post-translational modifications of tau protein: Implications for Alzheimer’s disease. Neurochem Int 58: 458–471. 21215781 10.1016/j.neuint.2010.12.023

[15] Lee MS, Tsai LH (2003) Cdk5: One of the links between senile plaques and neurofibrillary tangles? J Alz Dis 5: 127–137. 10.3233/jad-2003-5207 12719630

[16] Humbert S, Dhavan R, Tsai L (2000) p39 activates cdk5 in neurons, and is associated with the actin cytoskeleton. J Cell Sci 113: 975–983. 10683146 10.1242/jcs.113.6.975

[17] Dekosky ST, Scheff SW (1990) Synaptic loss in frontal cortex biopsies in ad correlation with cognitive severity. Ann Neurol 27: 457–464. 2360787 10.1002/ana.410270502

[18] Masliah E, Mallory M, Alford M, De Teresa R, Hansen LA, et al (2001) Altered expression of synaptic protein occurs early progression of Alzhiemer’s disease. Neurology 56: 127–129. 11148253 10.1212/wnl.56.1.127

[19] Kubavat AH, Khippal N, Tak S, Rijhwani P, Bhargava S, et al.. (2013) A randomized, comparative, multicentric clinical trial to assess the efficacy and safety of zileuton extended-release tablets with montelukast sodium tablets in patients suffering from chronic persistent asthma. Am. J. Ther. 20(2): 154–162. 10.1097/MJT.0b013e318254259b 22926233

